# Protective role of colitis in inflammatory arthritis *via* propionate-producing *Bacteroides* in the gut

**DOI:** 10.3389/fimmu.2023.1064900

**Published:** 2023-01-30

**Authors:** Hoh-Jeong Shon, Yu-Mi Kim, Kyeong Seog Kim, Jin-Ouk Choi, Sang-Hyun Cho, Sujin An, Se-Hyeon Park, Yong-Joon Cho, Joo-Hong Park, Sang-Uk Seo, Joo-Youn Cho, Wan-Uk Kim, Donghyun Kim

**Affiliations:** ^1^ Department of Biomedical Sciences, Seoul National University College of Medicine, Seoul, Republic of Korea; ^2^ Department of Microbiology and Immunology, Seoul National University College of Medicine, Seoul, Republic of Korea; ^3^ Center for Integrative Rheumatoid Transcriptomics and Dynamics, The Catholic University of Korea, Seoul, Republic of Korea; ^4^ Department of Biomedicine & Health Sciences, The Catholic University of Korea, Seoul, Republic of Korea; ^5^ Department of Clinical Pharmacology and Therapeutics, Seoul National University College of Medicine, Seoul, Republic of Korea; ^6^ School of Biological Sciences, Seoul National University, Seoul, Republic of Korea; ^7^ Institute for Basic Science, Seoul, Republic of Korea; ^8^ Department of Molecular Bioscience, College of Biomedical Science, Kangwon National University, Chuncheon, Republic of Korea; ^9^ Department of Microbiology, College of Medicine, The Catholic University of Korea, Seoul, Republic of Korea; ^10^ Division of Rheumatology, Department of Internal Medicine, the Catholic University of Korea, Seoul, Republic of Korea; ^11^ Institute of Endemic Diseases, Seoul National University Medical Research Center, Seoul, Republic of Korea

**Keywords:** arthritis, colitis, gut microbiota, Bacteroides, propionate

## Abstract

**Objectives:**

To investigate whether and how inflammatory disease in the intestine influences the development of arthritis, considering that organ-to-organ communication is associated with many physiological and pathological events.

**Methods:**

First, mice were given drinking water containing dextran sodium sulfate (DSS) and then subjected to inflammatory arthritis. We compared the phenotypic symptoms between the cohoused and separately-housed mice. Next, donor mice were divided into DSS-treated and untreated groups and then cohoused with recipient mice. Arthritis was then induced in the recipients. The fecal microbiome was analyzed by 16S rRNA amplicon sequencing. We obtained type strains of the candidate bacteria and generated propionate-deficient mutant bacteria. Short-chain fatty acids were measured in the bacterial culture supernatant, serum, feces, and cecum contents using gas chromatography-mass spectrometry. Mice fed with candidate and mutant bacteria were subjected to inflammatory arthritis.

**Results:**

Contrary to expectations, the mice treated with DSS exhibited fewer symptoms of inflammatory arthritis. Intriguingly, the gut microbiota contributes, at least in part, to the improvement of colitis-mediated arthritis. Among the altered microorganisms, *Bacteroides vulgatus* and its higher taxonomic ranks were enriched in the DSS-treated mice. *B. vulgatus, B. caccae*, and *B. thetaiotaomicron* exerted anti-arthritic effects. Propionate production deficiency further prevented the protective effect of *B. thetaiotaomicron* on arthritis.

**Conclusions:**

We suggest a novel relationship between the gut and joints and an important role of the gut microbiota as communicators. Moreover, the propionate-producing *Bacteroides* species examined in this study may be a potential candidate for developing effective treatments for inflammatory arthritis.

## Introduction

Organ-to-organ communication has evolved concomitantly in multicellular organisms to adjust to the external environment and maintain homeostasis (1). The interplay between organs is involved in many physiological and pathological events in the body, such as the adaptive response to external factors, maintenance of energy homeostasis, and migration of cells to target sites (1). The gastrointestinal system influences bodily functions that extend beyond traditional digestive functions (2, 3). The condition of the intestines directly influences the optimal function of other organs, which has yielded the concept of organ-to-organ communication, such as that between the gut and brain, lungs, and heart (2, 4, 5). The communication is mediated by hormones, cytokines, chemokines, and adhesion molecules (1, 4, 5). The gut microorganisms residing in the intestinal tract are involved directly and indirectly in interactions with other organs through various routes including the immune system, tryptophan metabolism, and vagus nerve and enteric nervous system (4–7). Gut microbes and their metabolites, such as short-chain fatty acids (SCFAs), branched-chain amino acids, and peptidoglycans, may contribute to the crosstalk between the gut and other organs by migrating to other parts of the body. This yields beneficial or pathological effects in target organs (2, 7, 8). Despite the increasing interest in organ-to-organ communications, the various forms and their mechanisms remain to be elucidated.

Concomitant diseases, in which each disease develops in different organs and tissues, can have a positive or negative relationship with each other (1, 9) but may result in poor organ-to-organ communication. For example, 36–46% of patients with inflammatory bowel disease (IBD) have at least one extraintestinal symptom, and rheumatic manifestations are the most frequent and comprise peripheral and axial joint involvement (10, 11). Peripheral arthritis is frequently found in Crohn’s disease (CD) and ulcerative colitis (UC), with a prevalence ranging from 17% to 20%. It mainly affects the large joints of the lower limbs, and less frequently those of the upper limbs (10). Axial involvement is more prevalent in patients with CD than in those with UC. It can include symptomatic or asymptomatic sacroiliitis with or without ankylosing spondylitis (10, 11).

Several genetic and nongenetic factors related to the development of colitis may contribute to the risk of inflammatory arthritis (9). The leaky gut of patients with IBD may allow the entry of gut microbiota from the lumen into the host and cause other problems in disseminated organs, such as joints, spine, skin, and eye (12). In addition, certain gut microbes are involved in the initiation and progression of inflammation-driven arthritis (13–15). However, the pathogenesis of rheumatic manifestations in patients with IBD has not yet been clarified. There is not yet direct evidence on the causality between IBD and inflammatory arthritis; thus, it remains unclear whether IBD and its associated changes in the microbiome accelerate or decelerate the progression of arthritis. Here, we investigated the influence of gut conditions on disease pathogenesis in joints using a colitis model, DSS-induced colitis, which is the most popular model in IBD research (16). We also used two animal models of inflammatory arthritis. Next, we examined whether the gut microbiota was involved in the influence of colitis on inflammatory arthritis. To identify bacterial candidates and their underlying mechanisms involved in the communication between the gut and joints, we performed 16S rRNA amplicon next generation sequencing (NGS) and *in vivo* experiments using mutant and type strains of bacteria.

## Materials and methods

### Mice

C57BL/6 female mice were purchased from Orientbio (Korea) and housed under specific pathogen free (SPF) or semi-SPF conditions in animal facilities at Seoul National University College of Medicine and the Catholic University of Korea. The mice were randomly allocated and raised in their appointed cages. All animal experiments were performed in accordance with protocols approved by the Institutional Animal Care and Use Committee at Seoul National University (SNU-181128-2-6, SNU-201008-2-2, and SNU-211129-2-2), and the Catholic University of Korea (CUMS-2016-0227-03, CUMS-2018-0318-01, and CUMS-2021-0080-20).

### Culture and preparation of *B. vulgatus*, *B. caccae*, and *B. thetaiotaomicron*

Type strains of *B. vulgatus* (NCTC 11154, cat. # ATCC 8482), *B. caccae* (VPI 3452A, cat# ATCC 8482) and *B. thetaiotaomicron* (VPI 5482, cat# KCTC 5723) were purchased or obtained from the American Type Culture Collection (Manassas, VA, USA) and Korean Collection for Type Cultures (Korea). *Bacteroides* were grown on brain heart infusion (BHI; BD Difco) agar plates supplemented with 5% sheep blood (cat# MB-S1876, MBcell, Korea) or in BHI broth supplemented (BHIS) with 5 mg/mL of hemin, 1 g/L of L-cysteine, 1 mg/L of resazurin and 1 g/L of NaHCO_3_ (Sigma). A single colony on the culture plate was transferred to 5 mL of BHIS broth and incubated in an anaerobic chamber (COY) for 16 h at 37 °C (17). These stationary phase cultures were diluted to 1:50 (*B. vulgatus* and *B. caccae*) or 1:20 (*B. thetaiotaomicron*) in 5 mL of fresh BHIS broth and then cultured anaerobically at 37 °C until the bacteria were at a concentration of 1 × 10^10^ CFU/mL (*B. vulgatus* and *B. caccae*) or 1 × 10^11^ CFU/mL (*B. thetaiotaomicron*). The bacterial culture supernatants obtained by centrifugation were used to analyze SCFAs, and the bacterial pellets were washed and resuspended in phosphate-buffered saline (PBS; Welgene, Korea) for the animal experiments.

### Generation of a *B. thetaiotaomicron* propionate-deficient mutant

The BT1686-1689 genes of *B. thetaiotaomicron* putatively encoding the four subunits of methyl malonyl-CoA transcarboxylase were deleted to block conversion of succinate into propionate (18). This was achieved with pLGB30 vectors according to a previously described deletion strategy (19). The upstream and downstream regions of the target locus were PCR-amplified using Phusion Hot Start II High-fidelity PCR Master Mix (cat.#F565, Thermo Fisher Scientific) and the following primer sets: BT1686-89_upstream, 5 ′-GCA TTA TGA GTG GAT CCC CCC ACA TGA ACA GCA TGC TTA TC-3′ (forward) and 5′-TAT TTT GTC CGA GTT CTT GTT GTT TTT TTA GAG TTT ATG-3′ (reverse); BT1686-89_downstream, 5′-ACA AGA ACT CGG ACA AAA TAT TAG GTG CGG-3′ (forward) and 5′-ATA TCG AAT TCC TGC AGC CCA TAG CTG ATA CCC AGA TAA TAA CAG-3′ (reverse). The PCR products were cloned into the SmaI site of the suicide vector pLGB30 using NEBuilder HiFi DNA Assembly (#E2621, NEB) according to the manufacturer’s instructions. The recombinant plasmid was transformed into the *E. coli* strain S17-λ*pir* for conjugal transfer into *B. thetaiotaomicron*. The deletions of the target genes were confirmed by a PCR analysis of bacterial genomic DNA.

### DSS-induced colitis and CFA (complete freund’s adjuvant)-induced arthritis model

Mice were *ad libitum* given fresh water (vehicle group) or water bottles containing DSS (DSS group) for 5 days (**Figure S1**). Then, the water bottles were replaced with fresh water and, after anesthetizing the mice, 20 μL of CFA (containing 2 mg/mL of heat-killed *Mycobacterium tuberculosis*; Chondrex) and IFA (Chondrex) were subcutaneously injected into each footpad (**Figure S1**). The swelling of the footpads and ankles was measured using calipers at indicated time points. After euthanization, the CFA-injected limbs were used to obtain macroscopic and hematoxylin and eosin (H&E)-staining images. In addition, body weights and stool states were monitored until the end of the study. The disease activity index (DAI) was calculated by scoring body weight loss, bloody stool, and stool consistency (**Table S1**).

### DSS-induced colitis and mBSA/IL-1β-induced arthritis model

Mice were *ad libitum* provided with fresh water (vehicle group) or 2.5% DSS water bottles (DSS group) for 5 days. Then, the water bottles were replaced with fresh water and, after anesthetizing the mice, 200 μg of methylated bovine serum albumin (mBSA, Sigma) was intra-articularly injected into the bilateral knee joints. The next day, recombinant human IL-1β (250 ng; R&D Systems) was subcutaneously administered into the left hind footpads, and the injection was repeated on the following two days. Seven days after the mBSA injections, the mice were euthanized, and tissues containing the knee joints were collected for H&E staining.

### Comparison of cohousing and separate housing

Mice were provided with fresh water bottles (vehicle group) or water bottles containing DSS (DSS group) for 5 days. Then, the water bottles were replaced with fresh water and, after anesthetizing the mice, CFA and IFA were subcutaneously injected into each footpad of all the mice, and the control and colitis groups were raised in the same cages or separately raised in divided cages until the end of the study (**Figure S2A**). Footpad and ankle swellings were measured using calipers at indicated time points.

### Transfer of gut microbes using coprophagy

The mice were divided into donor and recipient groups (**Figure S2B**). To reduce the number of preexisting gut microbes, the recipient group was provided with water bottles containing an antibiotic cocktail (1 g/L of ampicillin, 1 g/L of neomycin, 1 g/L of metronidazole, and 0.5 g/L of vancomycin) for 2 weeks before cohousing. The donor mice were divided into control and colitis groups. To transfer gut microbes from the donor to recipient mice, the mice in each donor group were cohoused with the recipient mice after 5 days of the DSS treatment (**Figure S2B**). Two days later, CFA and IFA were injected into the footpads of the recipient mice. Footpad and ankle swellings in the CFA-injected limbs were measured, and feces were collected at indicated time points (**Figure S2B**).

### Gavage experiment using *Bacteroides*

The mice were provided with water bottles containing an antibiotic cocktail *ad libitum* for 14 days. After ceasing the antibiotics, the mice were repeatedly administered *Bacteroides* species (1 × 10^9^ CFU/mouse) or PBS (200 μL; vehicle) at indicated time points. Four days after ceasing the antibiotics, CFA and IFA were subcutaneously injected into each footpad. Footpad and ankle swellings in the CFA-injected limbs were measured, and feces were collected at indicated time points.

### Hematoxylin and eosin staining

The CFA-injected hind limbs and mBSA/IL-1β-induced knee joints were fixed in 4% paraformaldehyde (Biosesang, Korea), decalcified in a decalcifying solution (Sigma) for 36 h, and embedded in paraffin. Sections (4-μm thick) were cut in the sagittal plane and stained with H&E. The slides were scanned using a Pannoramic MIDI digital slide scanner (3DHISTECH). Images were extracted using the Case Viewer software (3DHISTECH). The joint sections were cut at five depths that were approximately 100 μm apart. The slides were qualitatively evaluated using the following parameters: synovial hyperplasia, inflammation, and bone destruction. Disease severity was graded histologically from 0 (normal) to 3 (severe) by an investigator blinded to the experimental groups (20).

### Microbiome analysis

Total bacterial genomic DNA was extracted from feces using a stool DNA kit (Omega Bio-Tek). The Illumina 16S metagenomic sequencing library preparation protocol was then used to prepare 16S ribosomal RNA amplicons. The library was sequenced using the Illumina MiSeq system on a 250 bp paired-end platform. The obtained data were analyzed using QIIME 2 (version 2020.8). Linear discriminant analysis [LDA] effect size (LEfSe) analysis was performed using the online-based Galaxy server (https://huttenhower.sph.harvard.edu/galaxy/).

### Quantitative analysis of short-chain fatty acids

The concentrations of SCFAs were analyzed in the feces, cecum, and bacterial culture supernatant samples as previously described (21). In brief, water was added to the feces and cecum at a 50 mg: 500 µL ratio, after which it was homogenized by vortexing for 20 min. The mixture was centrifuged for 5 min at 18,341 × *g* and 4°C, and the supernatants were transferred into new plastic tubes. Next, 10 µL of 1.0 M hydrochloric acid and 20 µL of internal standard solution (500 µg/mL acetic acid-d_4_ and 30 µg/mL butyric acid-d_7_) were added to each 100 µL of fecal homogenate, cecal homogenate and bacterial supernatant. SCFAs were extracted with 200 µL of methyl tert-butyl ether (MTBE). The MTBE phase was then transferred into an autosampler vial and analyzed by gas chromatography-mass spectrometry (Agilent 7890B gas chromatography coupled with Agilent 7000B mass spectrometer, Agilent Technologies Inc., Santa Clara, CA, USA). Ions at 60.0 m/z were monitored for acetic acid, butyric acid, isovaleric acid and valeric acid; 63.0 m/z for acetic acid-d_4_ and butyric acid-d_7_; 73.0 m/z for isobutyric acid; and 74.0 m/z for propionic acid. A calibration curve was constructed to quantify the SCFAs by plotting the peak area ratio of each SCFA to the corresponding internal standard (acetic acid-d_4_ for acetic acid and butyric acid-d_7_ for propionic acid, butyric acid, valeric acid, isobutyric acid, and isovaleric acid) versus the concentration of each SCFA. Data were acquired and analyzed using the Masshunter quantitative program B.06.00 (Agilent Technologies Inc., Santa Clara, CA, USA).

### IL-10 expression in the spleen and mesenteric lymph nodes

Splenocytes and lymphocytes were collected by mashing the spleen and mesenteric lymph nodes using a 70-μm cell strainer (BD Falcon). Red blood cells (RBCs) were lysed following the addition of cold RBC Lysis Buffer (eBiosciences) for 5 min. Splenocytes and lymphocytes (1 × 10^6^ cells/well in a 96 well-plate) were treated with 50 ng/mL of Phorbol myristate acetate (PMA; Sigma) and 1 μg/mL of ionomycin (Sigma). After 24 h, the supernatants were collected, and the levels of IL-10 were measured using an ELISA kit (R&D Systems) according to the manufacturer’s instructions.

### Data access

The 16S rRNA amplicon raw data were deposited in the SRA database (accession code PRJNA869574).

### Statistical analyses

General statistical analyses were performed using the Prism software (GraphPad Software, version 8.02). We examined differences in the results between groups of individual animals using the Mann–Whitney test (between two groups) and Kruskal–Wallis test, followed by Dunn’s multiple comparisons test (between three groups). No samples or animals were excluded from the analyses, except for dead individuals. Differences were considered statistically significant at P < 0.05.

## Results

### Colitis protects from inflammatory arthritis

**Figure 1:**
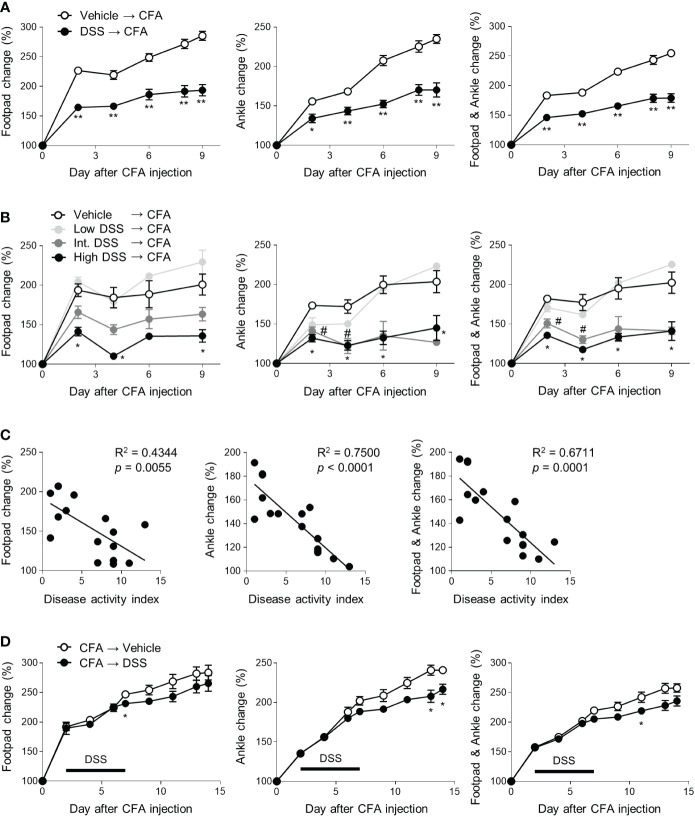
Protective effects of colitis on CFA-induced arthritis. **(A–C)** Mice (*n* = 5 or 4 per group) were provided with drinking water with or without DSS for 5 days. CFA and IFA (20 μL) were then subcutaneously injected into each footpad. The swelling at the footpads and ankles was measured at indicated time points after the injections **(C)** The correlation between the DAI score and swelling at footpads and ankles of the mice on day 9 among data in the **(B)**. **(D)** Mice (*n* = 5 per group) were subcutaneously treated with CFA and IFA, and 2 days later, given drinking water containing DSS or fresh water for 5 days (marked as a bar). The concentration of DSS was 2.5% **(A, D)** and Low DSS, Int. DSS, and High DSS indicated 2, 2.5, and 3% concentration, respectively **(B)**. The results are representative of at least two independent experiments. The data are shown as means ± SEM. *P < 0.05 and **P < 0.01 with the Mann–Whitney test **(A, D)**. *P < 0.05 (between the vehicle and DSS groups or high DSS groups) and #P < 0.05 (between the vehicle and intermediate DSS groups) with the Mann–Whitney test **(B)**.

To investigate the effects of colitis on inflammatory arthritis, the mice were given water bottles with or without DSS *ad libitum*. CFA and IFA were then subcutaneously injected into their footpads, and the thickness of their footpads and ankles was monitored (**Figure S1**). Contrary to our expectations, the footpads and ankles of the DSS-pretreated mice were less swollen in the CFA-injected limbs than those of the control group (**Figure 1A**). To confirm the protective effects of colitis on arthritic symptoms, the mice were divided into 0, 2, 2.5 and 3% DSS-treated groups. All groups were administered CFA and IFA. Edema at the footpads and ankles following the CFA injections was ameliorated by DSS in a dose-dependent manner and most distinctly suppressed by high doses of DSS (**Figure 1B**). Moreover, the arthritic symptoms of the individual mice were inversely proportional to the severity of their colitis (**Figure 1C**). DSS-induced colitis therefore exerts a preventive effect on the development of inflammatory arthritis. Next, we switched the order of the induction of colitis and arthritis. Post-treatment with DSS significantly suppressed the CFA-induced swelling of the footpads and ankles, suggesting that colitis exerts a therapeutic effect on arthritic symptoms (**Figure 1D**). To examine whether the protective effects of colitis were limited to a specific model of CFA-induced arthritis, we used the mBSA/IL-1β-induced arthritis model. Similar to the results in the CFA-induced arthritis model, colitis ameliorated the histopathological severity of knee joint arthritis caused by mBSA and recombinant IL-1β (**Figure S3**). Inducing colitis can therefore ameliorate the symptoms of inflammatory arthritis.

### Gut microbiota altered by colitis ameliorates inflammatory arthritis

**Figure 2:**
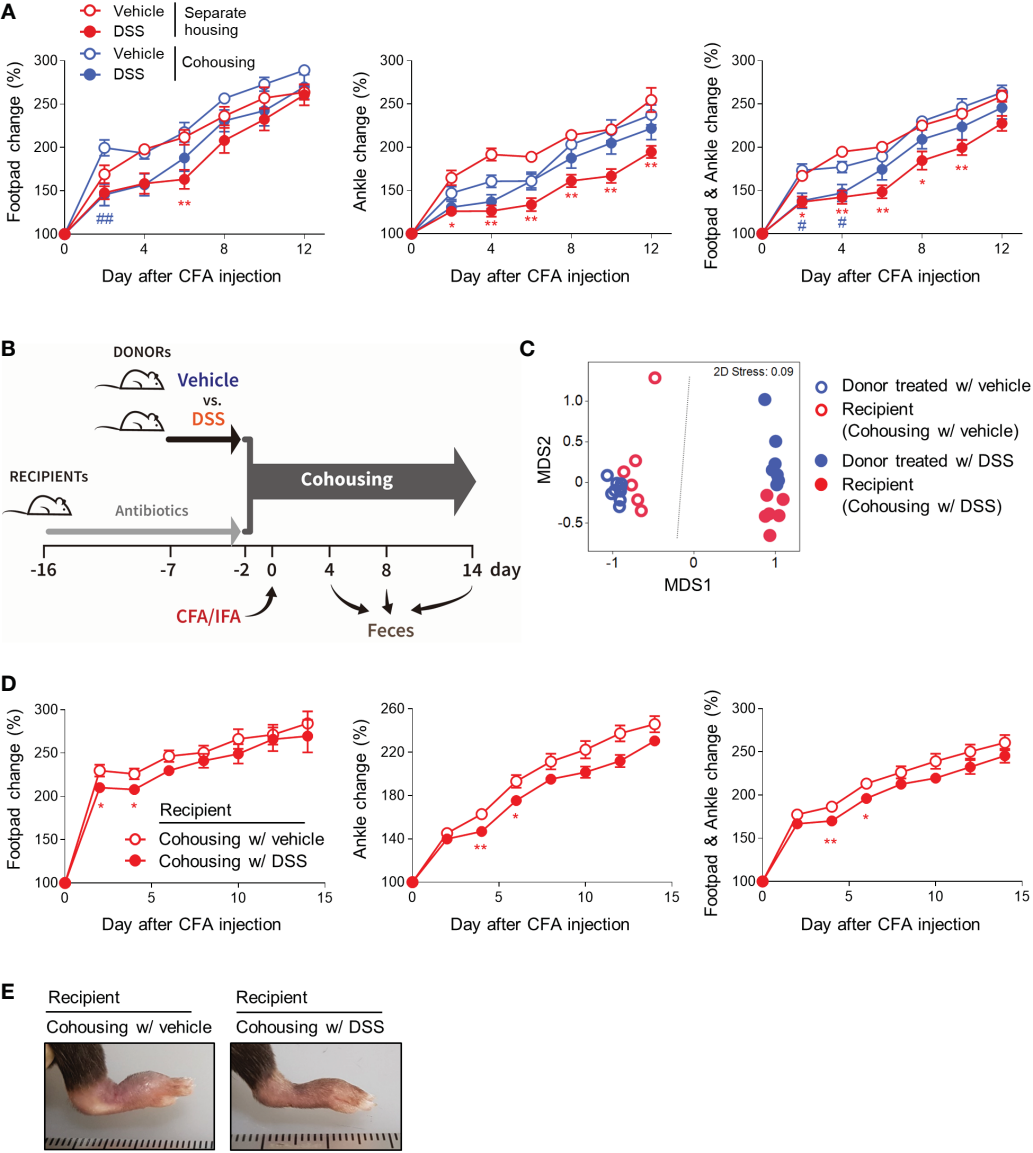
Gut microbiota contributes to colitis-mediated protection from arthritis. **(A)** Mice (*n* = 5 per group) were provided with fresh water or 2.5% DSS water bottles for 5 days. CFA and IFA were then subcutaneously injected into the footpads of all the mice. The control and colitis groups were mixed in the same cages or raised separately in the divided cages. The swelling at the footpads and ankles was then assessed. **(B–E)** Recipient mice (*n* = 6 per group) were given water bottles containing an antibiotic cocktail for 2 weeks before cohousing. Donor mice (*n* = 8 per group) were provided with fresh water or 2.5% DSS water bottles for 5 days before cohousing. The donor mice were cohoused with the recipient mice and, 2 days later, CFA and IFA were subcutaneously injected into each footpad of the recipient mice. **(B)** Experimental scheme. **(C)** Beta-diversity (non-metric multidimensional scaling [NMDS] plot) of the microbial communities on day 4. Each dot represents an individual mouse. **(D)** The swelling at the footpads and ankles of the recipient mice. **(E)** Representative macroscopic images of the CFA-injected limbs. The results are representative of at least two independent experiments. The data are shown as means ± SEM. *P < 0.05 and **P < 0.01 (between separately-housed groups) or #P < 0.05 and ##P < 0.01 (between cohoused groups) with the Mann–Whitney test **(A)**. *P < 0.05 and **P < 0.01 with the Mann–Whitney test **(D)**.

Given the coprophagy observed in rodents and other animals, cohousing transfers the gut microbiota from one mouse to the others in the same cage, which is widely accepted as a method for normalizing microbial communities (22). To examine the role of gut microbiota in colitis-induced protection against arthritis, we utilized cohousing. First, we compared arthritic symptoms when the DSS-treated and untreated mice were raised in separate and the same cages (**Figure S2A**). In the cohoused mice, the protective effects of colitis on inflammatory arthritis decreased compared with those in the separately-housed mice (**Figure 2A**). To further confirm the importance of gut microbiota, we divided the mice into two donor and two recipient groups (**Figures S2B**, **2B**). The donor mice were provided water bottles with or without DSS for 5 days and then cohoused with each recipient group that had been pretreated with an antibiotic cocktail for 2 weeks before cohousing (**Figures S2B**, **2B**). After 2 days of cohousing, CFA and IFA were injected into the footpads of the recipients. The thickness of the footpads and ankles was then measured, and feces were collected (**Figures S2B**, **2B**). As expected, the beta-diversity, which describes differences in the gut microbiota between each pair of samples showed that DSS altered the gut microbiota and that the gut microbial compositions were similar within the donor and recipient mice raised in the same cage (**Figure 2C**). Consistent with the results from the comparison of cohousing and separate-housing, recipients cohoused with DSS-treated mice during the initial period of arthritis induction displayed less swelling at the footpads and ankles than recipients cohoused with the control (**Figures 2D, E**). Gut microbiota may, therefore at least partly, contribute to the beneficial effects of colitis on inflammatory arthritis.

### *B. vulgatus* is one of the gut microorganisms altered by DSS-induced colitis

**Figure 3:**
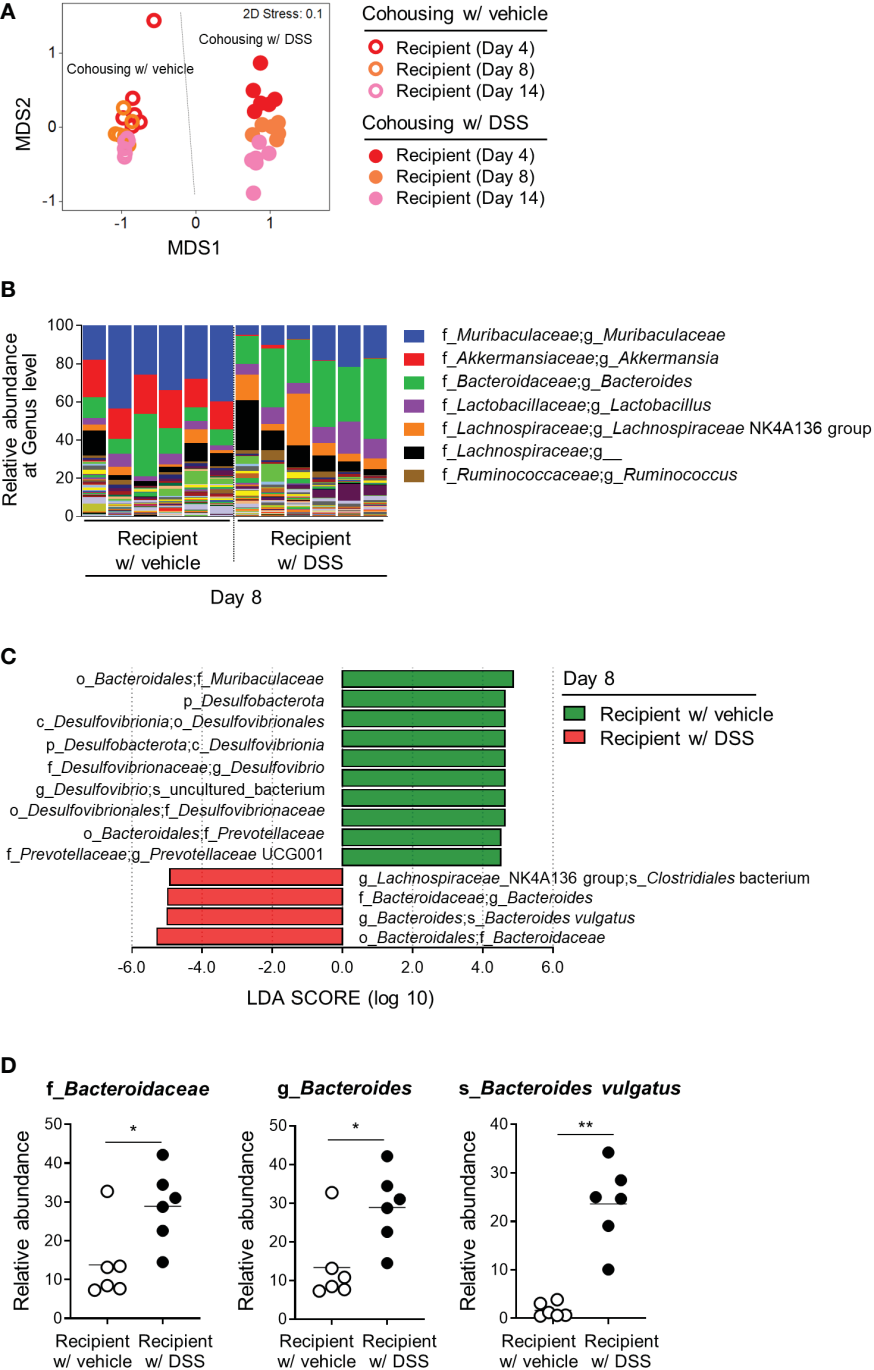
Gut microbial candidates involved in the colitis-mediated protection from arthritis. The gut microbiome obtained from the recipients in **Figure 2B** was analyzed. **(A)** Beta-diversity (NMDS plot) of the microbial communities of the recipient mice. Each dot represents an individual mouse. **(B)** Relative abundance of bacterial taxa at the genus level. The top seven taxa are listed in the right legend. **(C)** LEfSe analysis of the recipients cohoused with the vehicle and DSS-induced colitis groups (logarithmic LDA score > 4). Significant differences in taxa were obtained using the Kruskal–Wallis test (α < 0.05). **(D)** Among the bacterial taxa selected from the LEfSe analysis, the relative abundance of the family *Bacteroidaceae*, genus *Bacteroides*, and species *Bacteroides vulgatus* in each taxonomic rank are shown. Each dot represents an individual mouse and the means are displayed as a line. *P < 0.05 and **P < 0.01 with the Mann-Whitney test.

To identify bacteria that exert protective effects against inflammatory arthritis, feces obtained from the recipient mice on days 4, 8, and 14 after cohousing were analyzed (**Figure 2B**). There was no difference in the alpha-diversity of the microbial communities, as indicated by the Chao1, Simpson, and Shannon indices, on days 4 and 8 (**Figure S4A**). The control recipients on day 14 had more species, however, and fewer differences in the abundance of each species than the recipients cohoused with the DSS-treated donors (**Figure S4A**). Consistent with the data in **Figure 2C**, the recipients cohoused with the DSS-treated donors had different gut microbiomes compared to the recipients cohoused with the control, regardless of the collection time points (**Figure 3A**). After comparing the bacterial abundance in the recipients on day 8, the genera *Muribaculaceae* and *Akkermansia* were found to be abundant in the control recipients cohoused with vehicle donors, while the genera *Bacteroides* and *Lactobacillus* were enriched in the recipients cohoused with DSS-treated donors (**Figure 3B**). Additionally, an LEfSe analysis of the amplicon sequence variants (ASVs) revealed that the family *Muribaculaceae*, genera *Desulfovibrio* and *Prevotellaceae* uncultured group 001, and their associated taxonomic ranks were more abundant in the control recipients, while the family *Bacteroidaceae*, genus *Bacteroides*, and species *Bacteroides vulgatus* and *Clostridiales* bacterium were enriched in the recipients cohoused with DSS-treated donors (**Figure 3C**). Likewise, an increase in the genus *Bacteroides* and decrease in the family *Muribaculaceae* were found in fecal samples on days 4 and 14 (**Figures S4B–D**). Among the distinguishing bacteria between the recipient groups, the family *Bacteroidaceae*, genus *Bacteroides*, and species *B. vulgatus* increased substantially in the recipients cohoused with DSS-treated donors (**Figure 3D**), suggesting that *B. vulgatus* may be one of the bacterial candidates involved in colitis-induced protection from inflammatory arthritis.

### *B. vulgatus* prevents inflammatory arthritis

**Figure 4:**
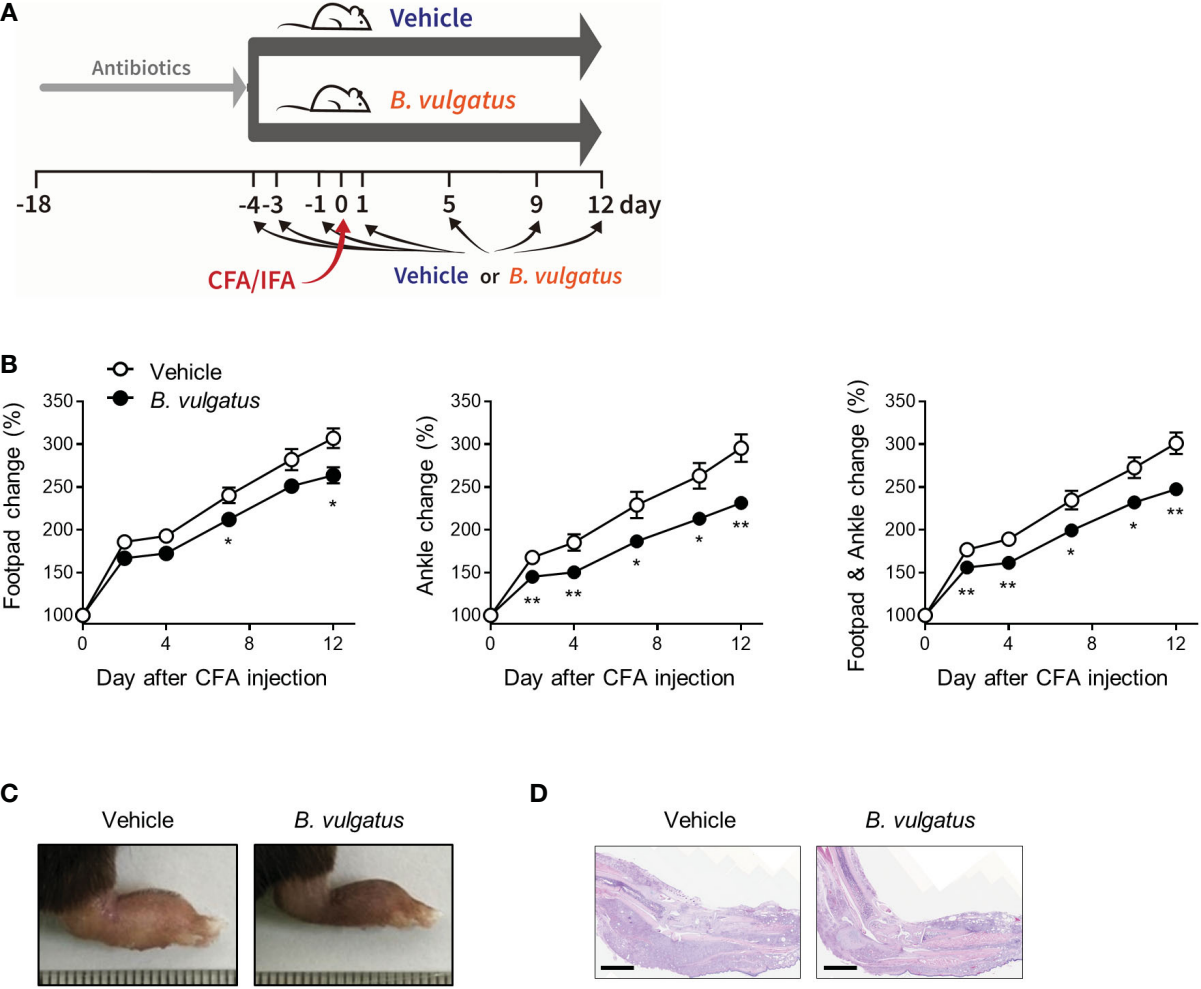
*B. vulgatus* protects from CFA-induced arthritis. Mice (*n* = 9 or 10 per group) were given drinking water containing an antibiotic cocktail for 14 days. After the cessation of the antibiotics, the mice were repeatedly administered *B*. *vulgatus* at indicated time points. Four days after the cessation of the antibiotics, CFA and IFA were injected into each footpad. **(A)** Experimental scheme. **(B)** The swelling at the footpads and ankles. The data are shown as means ± SEM. Representative macroscopic images **(C)** and H&E staining image **(D)** of CFA-injected limbs. Scale bar, 2 mm. The results are representative of at least two independent experiments. *P < 0.05 and **P< 0.01 with the Mann–Whitney test.

To examine whether *B. vulgatus* is beneficial in the pathogenesis of arthritis, the mice were repeatedly fed with a type strain of *B. vulgatus* or PBS (Vehicle) at indicated time points, and CFA and IFA were subcutaneously injected into each footpad (**Figure 4A**). *B. vulgatus* significantly suppressed the swelling of the ankles and footpads compared to the PBS-fed vehicle group (**Figures 4B, C**). Consistent with macroscopic pedal edema, the number of infiltrated inflammatory cells was reduced in the *B. vulgatus-*administered mice compared to that in the limbs of the vehicle group (**Figure 4D**). Thus, *B. vulgatus* may be an important contributor to colitis-mediated protection from inflammatory arthritis.

### DSS treatment and *B. vulgatus* induce the production of propionate

To understand the mechanisms underlying the beneficial effects of *B. vulgatus* and DSS-induced colitis in remote joint tissue, we assessed the expression of the anti-inflammatory cytokine IL-10. This was not only because IL-10 is an important contributor to immune homeostasis, but also because *B. fragilis*, another *Bacteroides* species, induces IL-10-secreting T cells (23). However, there were no differences in IL-10 expression in the splenocytes and mesenteric lymph node cells between *B. vulgatus*-administered and control mice, indicating that IL-10 may not be involved in the anti-inflammatory effects of *B. vulgatus* (**Figure S5**).

**Figure 5:**
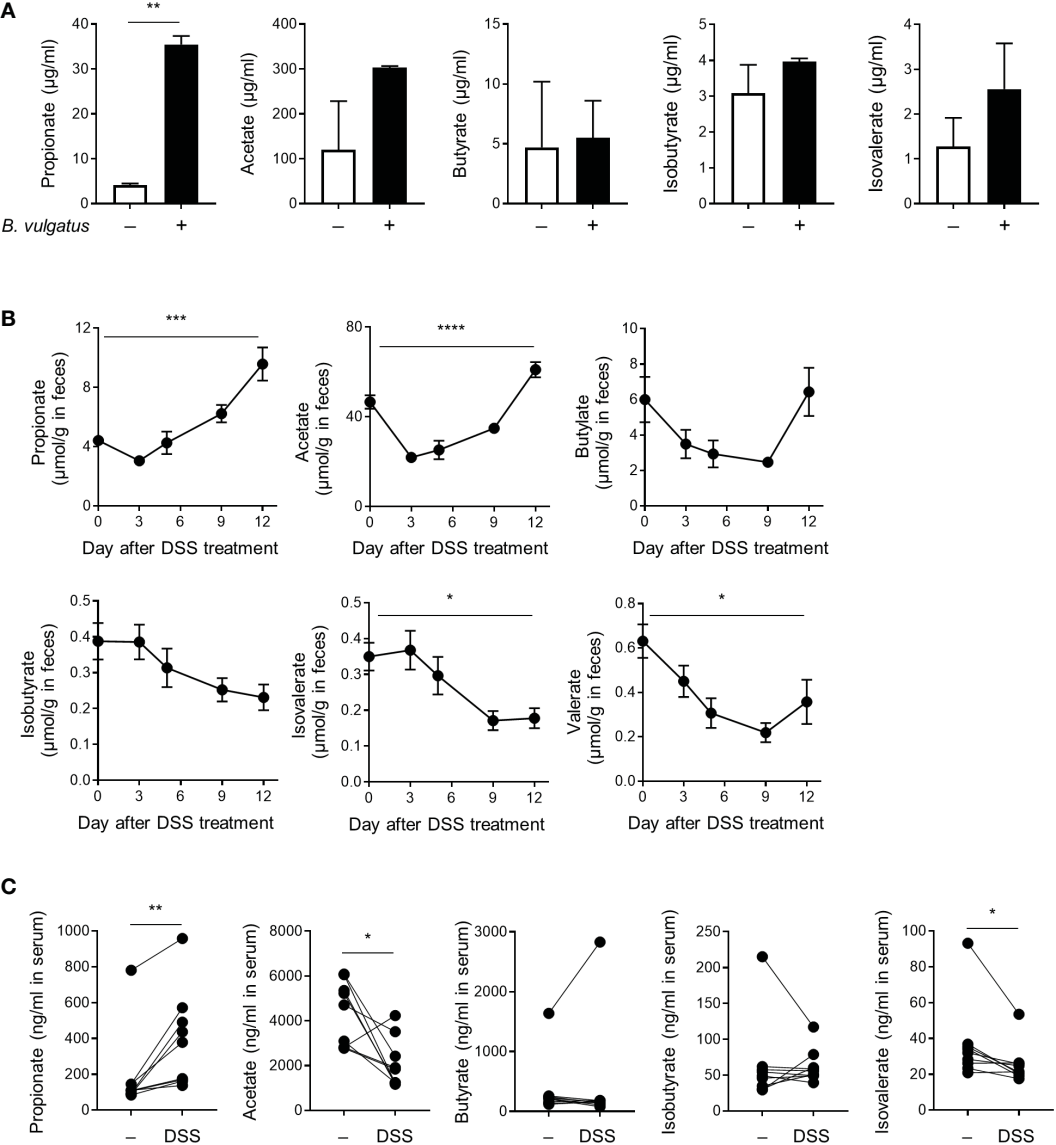
Production of short-chain fatty acids. **(A)** The amount of SCFAs secreted by *B*. *vulgatus* (2 × 10^9^ CFU/mL) during cultivation in BHIS broth. **P < 0.01 by an unpaired *t*-test. **(B, C)** Mice (*n* = 9) were provided with drinking water with DSS for 5 days, which was then replaced with fresh water. **(B)** The amount of SCFAs in the feces collected at indicated time points. The data are shown as means ± SEM. *P < 0.05, ***P < 0.001 and ****P < 0.0001 by a one-way RM ANOVA. **(C)** The amount of SCFAs in the serum collected before DSS-treatment and after 12 days of DSS-treatment. Each dot represents an individual mouse. *P < 0.05 and **P < 0.01 with a paired *t*-test.

SCFAs derived from the microbial fermentation of dietary fibers exert immune regulatory properties (24). Serum SCFAs correlate with the progression of arthritis in individuals at risk of rheumatoid arthritis (RA), and SCFA treatment inhibits the pathogenesis of experimental arthritis (25, 26). Therefore, we examined the level of SCFAs in the culture media of *B. vulgatus* and the sera, feces, and cecum contents of DSS-treated mice. *B. vulgatus* produced propionate and acetate, but butyrate, isobutyrate, isovalerate, and valerate were not detected (**Figure 5A** and data not shown). Moreover, DSS gradually increased the concentration of propionate in the feces and decreased the levels of isovalerate (**Figure 5B**). Similar changes were observed in the sera of the DSS-treated mice (**Figure 5C**). Other SCFAs, including acetate, butyrate, and isobutyrate, in the sera and feces did not show a consistent trend (**Figures 5B, C**). There were no significant changes in the SCFA levels in the cecum contents (**Figure S6**). Bacteria-derived propionate may therefore mediate the beneficial effects of colitis and *B. vulgatus* on inflammatory arthritis.

### Propionate-producing bacteria play a critical role in the protection from inflammatory arthritis

**Figure 6:**
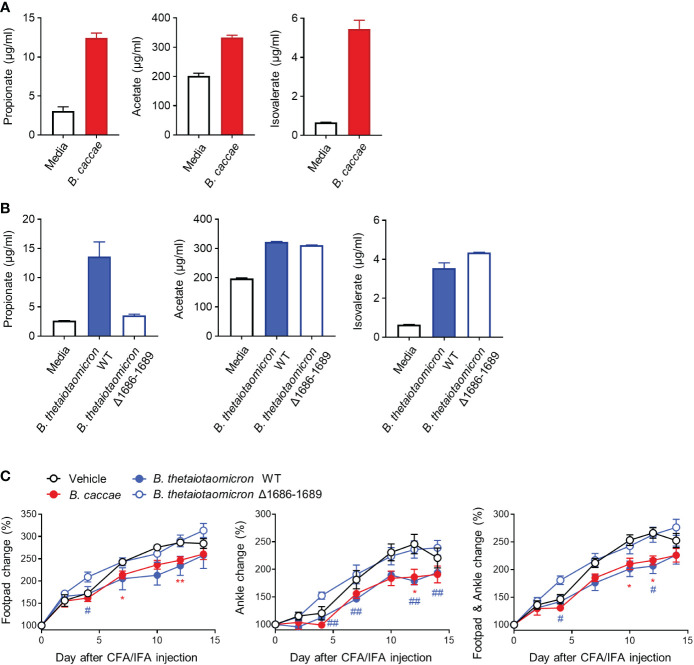
Other propionate-producing *Bacteroides* and mutant form and their protective effects on CFA-induced arthritis. The amount of SCFAs secreted by *B*. *caccae* **(A)** and *B. thetaiotaomicron* (WT and mutant Δ1686-1689) **(B)** (2 × 10^9^ CFU/mL) during cultivation in BHIS broth. **(C)** Mice (*n* = 5 per group) were intragastrically administered *B*. *caccae* and *B. thetaiotaomicron* (WT and mutant Δ1686-1689) and treated with CFA/IFA according to the same scheme in **Figure 4B**. The data are shown as means ± SEM. *P < 0.05 and **P < 0.01 (*B. caccae* to vehicle) or #P < 0.05 and ##P < 0.01 (*B. thetaiotaomicron* WT to vehicle) with the Mann–Whitney test.

*Bacteroides* are among the largest contributors to propionate formation within the human gut (27, 28). To investigate whether other *Bacteroides* and their propionate protect against inflammatory arthritis, we obtained other *Bacteroides* species, including *B. caccae* and *B. thetaiotaomicron*, and produced a mutant form (Δ1686-1689) of *B. thetaiotaomicron* with a defect in propionate production. As expected, *B. caccae* and *B. thetaiotaomicron* produced propionate, acetate, and isovalerate, but not butyrate or valerate, to varying extents (**Figures 6A, B** and data not shown). In contrast, the propionate-producing ability of *B. thetaiotaomicron* disappeared in the mutant form, despite the unabated production of acetate and isovalerate (**Figure 6B**). Similar to *B. vulgatus*, *B. caccae* and *B. thetaiotaomicron* significantly ameliorated edema at the footpads and ankles compared to the control (**Figure 6C**). However, the beneficial effects of *B. thetaiotaomicron* on arthritis disappeared in the mutant form of *B. thetaiotaomicron* (**Figures 6C**, **S7**). *Bacteroides*-producing propionate may therefore be responsible for the colitis-induced amelioration of inflammatory arthritis.

## Discussion

Organ-to-organ communication has been suggested as a gatekeeper of metabolic health (1) and regulator of lipid metabolism, tissue homeostasis, and protection (29–31). We explored whether IBD in the intestines directly worsens inflammatory arthritis in the joints and, if so, assessed potential messengers which mediate the interaction between the gut and joints. To accomplish this, we used DSS-induced colitis, CFA-induced arthritis, and mBSA/IL-1β-induced arthritis mouse models. Contrary to our expectations, DSS-induced colitis was negatively correlated with the severity of arthritic symptoms in our animal models. Thus, our first hypothesis was reconsidered. HLA-B27-associated human disorders and transgenic rats expressing HLA-B27 and human β2m have shown that B27 is indispensable for several concomitant diseases, such as ankylosing spondylitis, reactive arthritis, psoriatic arthritis, IBD, and uveitis (32). Similarly, many joint diseases associated with gastrointestinal complications can be attributed to genetic factors. The protective effects of colitis on arthritis may therefore not be an unusual phenotype, but rather a new finding, regardless of common genetic factors.

Most of the microorganisms that inhabit the body exist in the gastrointestinal tract (3). There is accumulating data on the signaling of gut microbes to extraintestinal organs that influence health and disease (3, 33). Gut microbiota plays a crucial role in the pathogenesis of inflammatory arthritis. For example, arthritic symptoms spontaneously develop in K/BxN, *IL1rn* ^−/−^, and SKG mice under specific pathogen-free conditions but not under germ-free (GF) conditions (13, 34, 35). In addition, the gut microbial composition of patients with RA differs from that of healthy individuals (13, 36). Various commensal bacteria, including segmented filamentous bacteria, *Lactobacillus bifidus* and *Prevotella copri*, contribute to the development and severity of arthritis, whereas others, including *Prevotella histicola*, ameliorate the severity of arthritis (13, 14, 34, 36). Here, we used the coprophagy of the mice to demonstrate that the gut microorganisms altered by IBD were attributable to the amelioration of inflammatory arthritis, indicating that the gut microbes act as messengers in the communication between the gut and joints. The altered microbiota further helped to maintain homeostasis in the joints, which may have resulted from the coevolution of host-microbiome system to protect the host from excessive inflammation. Microbiota can manipulate rapid phenotypic changes in hosts, which may be important for attuning to unpredictable or fluctuating environments, such as in the defense against pathogens (37). These interactions may be based on reciprocal selection between the host and its microbiota. However, further evidence is needed to corroborate whether and at what scale such selection can cause coevolution (37).

Identifying bacterial species or their metabolites is of even greater interest because it is difficult to precisely adjust the whole bacterial community, and treatment with antibiotics may result in adverse effects (38). We observed an increase in the abundance of *B. vulgatus* and its higher taxonomic ranks in the DSS-treated mice. Members of the genus *Bacteroides* are major commensals in healthy humans and gram-negative obligate anaerobes that colonize the colon (39). As commensals, *Bacteroides* generally play beneficial roles in the gut, such as inhibiting pathogen colonization and supplying nutrients to other commensal residents (39). Conversely, *Bacteroides* may act as pathobionts in genetic and environmental contexts (40). To our knowledge, however, *Bacteroides*-dominated feces or *B. vulgatus* have not previously been implicated in the pathogenesis of inflammatory arthritis, although a *Bacteroides*-dominated group has been found among patients with new-onset RA (13).

In this study, the mice were repeatedly administered pure cultures of *B. vulgatus, B. caccae*, and *B. thetaiotaomicron*, which led to reduced edema in their footpads and ankles. Several strains of probiotics, including *Lactobacillus* and *Bifidobacteria*, have been tested for their benefits in patients with RA (41). Although probiotic supplements can boost the effects of concomitant pharmacological treatments, evidence remains insufficient to make definite recommendations (41). Future studies should further investigate whether the beneficial effects of *Bacteroides* on RA are better than those of probiotics. However, because commensal *Bacteroides* are prevalent in the colon (39), administering *Bacteroides* species may be favorable for colonizing the intestinal tract, which affects their abundance in the gut. Moreover, because probiotic supplementation seems to have no clinically significant adverse effects (41), there is little concern regarding the side effects of *Bacteroides*. Thus, the three *Bacteroides* species examined in this study may serve as potential probiotics for the prevention and treatment of inflammatory arthritis.

SCFAs, which are primary gut bacterial products derived from dietary fibers, are beneficial for various physiological processes in the body and exhibit health-promoting properties in many inflammatory disorders, including colitis and arthritis (42, 43). Bacteria-derived SCFAs allow communication with peripheral organs and tissues in the host *via* multiple mechanisms, including histone deacetylase inhibition, G-protein-coupled receptor signaling, and energy source supplies (8, 43). Small amounts of SCFAs (mostly acetate and possibly propionate) move to target organs at remote sites and yield direct beneficial effects (43). Although a wide range of pre-clinical evidence has supported the role of SCFAs as modulators of joint inflammation, there are no reports of using SCFAs for arthritis in clinical trials. Instead, high-fiber dietary intervention has been found to increase SCFA levels and decrease the levels of pro-inflammatory cytokines and bone erosion markers in patients with RA (42, 44). A high-fiber diet leads to a predominance of the phylum *Bacteroidetes* in mice with collagen-induced arthritis and promotes the concomitant production of propionate, resulting in the improvement of arthritis and bone erosion (45). In addition, propionate suppresses the induction of inflammatory mediators and induces cellular senescence in synovial fibroblasts (46). The addition of propionate to drinking water improves arthritic symptoms in mice with collagen- and mBSA/MSU-induced arthritis (45, 46). Here, we found that DSS increased propionate levels in the blood and feces, whereas the *B. thetaiotaomicron* mutant with a defect in propionate production lost its beneficial effects on inflammatory arthritis. Thus, propionate production may be an important mechanism underlying the improvement of inflammatory arthritis upon treatment with DSS or *Bacteroides*. Further studies are required to elucidate the molecular mechanistic gaps between propionate production and joint inflammation.

Despite our findings, this study had several limitations. First, here, we did not consider the gut microbial composition in the process of epithelial repair and wound healing after ceasing DSS-treatment. Because their effect on the inflammatory arthritis may differ from our results, more exquisite experimental design would be needed in the further study. Second, commensal species and mechanisms that were not identified in this study may also contribute to the observed effects on the progression of inflammatory arthritis. Third, our results do not support a bidirectional relationship between the gut and joints. Future studies should therefore investigate whether the conditions of the joints influence the state of the intestines. Finally, because numerous studies have shown that the beneficial effects of probiotics depend on the strain (41), we should attempt to identify more effective stains than the type strains of the three *Bacteroides* species for the treatment of inflammatory arthritis.

In conclusion, our study provides novel insights into the gut-joint axis and highlights the importance of the gut microbiota as nongenetic factors related to the pathogenesis of arthritis and messengers between the gut and joints. Moreover, the protective effects of *Bacteroides* and propionate noted in this study may provide a basis for developing potent probiotics and postbiotics for the prevention or treatment of inflammatory arthritis.

## Data availability statement

The datasets presented in this study can be found in online repositories. The names of the repository/repositories and accession number(s) can be found below: https://www.ncbi.nlm.nih.gov/bioproject/PRJNA869574/.

## Ethics statement

The animal study was reviewed and approved by Institutional Animal Care and Use Committee (IACUC) at Seoul National University: IACUC at the Catholic University of Korea.

## Author contributions

DK contributed Conceptualization, Formal analysis, Investigation, Writing – Original Draft, Writing – Review & Editing, Visualization, Supervision, Project administration and Funding acquisition. W-UK contributed Conceptualization, Writing – Review & Editing, Supervision, Project administration and Funding acquisition. H-JS contributed Methodology, Validation, Investigation and Writing – Original Draft. Y-MK contributed Methodology, Investigation, Validation, Writing – Original Draft, and Funding acquisition. KK analyzed Investigation, Validation, and Writing – Original Draft. J-OC contributed Validation and Investigation. S-HC contributed Investigation. SA contributed Investigation. S-HP contributed Investigation. Y-JC contributed Software, Data Curation, and Writing - Original Draft. J-HP contributed Writing – Original Draft and Supervision. S-US contributed Investigation. J-YC contributed Writing - Original Draft and Supervision. All authors contributed to the article and approved the submitted version.
