# Supplementary material for: Protective role of colitis in inflammatory arthritis via propionate-producing Bacteroides in the gut
Source: Front Immunol. 2023 Jan 30;14:1064900. doi: 10.3389/fimmu.2023.1064900 (PMC9923108; doi:10.3389/fimmu.2023.1064900)
Supplement: Supplementary file 1 [file DataSheet_1.pdf]

Supplemental material

Protective Role of Colitis in Inflammatory Arthritis via Propionate-Producing *Bacteroides* in the Gut

Hoh-Jeong Shon[1,2,*], Yu-Mi Kim[3,4,*], Kyeong Seog Kim[1,5], Jin-Ouk Choi[1,2], Sang-Hyun Cho[6], Sujin An[1,2], Se-Hyeon Park[3,4], Yong-Joon Cho[6,7,8], Joo-Hong Park[6], Sang-Uk Seo[9], Joo-Youn Cho[1,5], Wan-Uk Kim[3,4,10], and Donghyun Kim[1,2,11]

[1] *Department of Biomedical Sciences, Seoul National University College of Medicine, Seoul, Republic of Korea*
[2] *Department of Microbiology and Immunology, Seoul National University College of Medicine, Seoul, Republic of Korea*
[3] *Center for Integrative Rheumatoid Transcriptomics and Dynamics, The Catholic University of Korea, Seoul, Republic of Korea*
[4] *Department of Biomedicine & Health Sciences, The Catholic University of Korea, Seoul, Republic of Korea*
[5] *Department of Clinical Pharmacology and Therapeutics, Seoul National University College of Medicine, Seoul, Republic of Korea*
[6] *School of Biological Sciences, Seoul National University, Seoul, Republic of Korea*
[7] *Institute for Basic Science, Seoul, Republic of Korea*
[8] *Department of Molecular Bioscience, College of Biomedical Science, Kangwon National University, Chuncheon, Republic of Korea*
[9] *Department of Microbiology, College of Medicine, The Catholic University of Korea, Seoul, Republic of Korea*
[10] *Division of Rheumatology, Department of Internal Medicine, the Catholic University of Korea, Seoul, Republic of Korea*
[11] *Institute of Endemic Diseases, Seoul National University Medical Research Center, Seoul, Republic of Korea*

* Hoh-Jeong Shon and Yu-Mi Kim contributed equally to this study.

Correspondence to Donghyun Kim, PhD ＆ Wan-Uk Kim, MD PhD

List of Supplemental material

Figure S1
Figure S2
Figure S3
Figure S4
Figure S5
Figure S6
Figure S7
Table S1

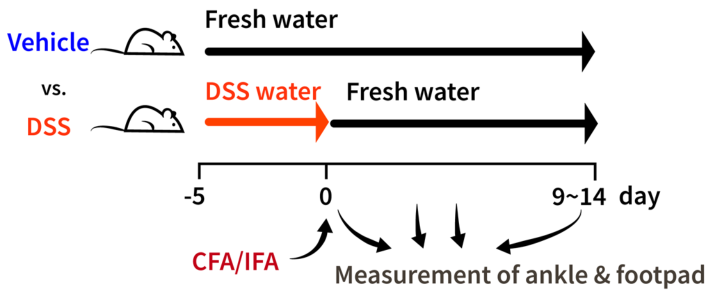

Figure S1 Experimental scheme for examining the effect of DSS-induced colitis on CFA-induced arthritis. Mice were provided with fresh water or DSS water bottles for 5 days. The water bottles were replaced with fresh water bottle and CFA and IFA were then subcutaneously injected into the footpads of all the mice. The swelling of the footpads and ankles was measured at indicated time points.

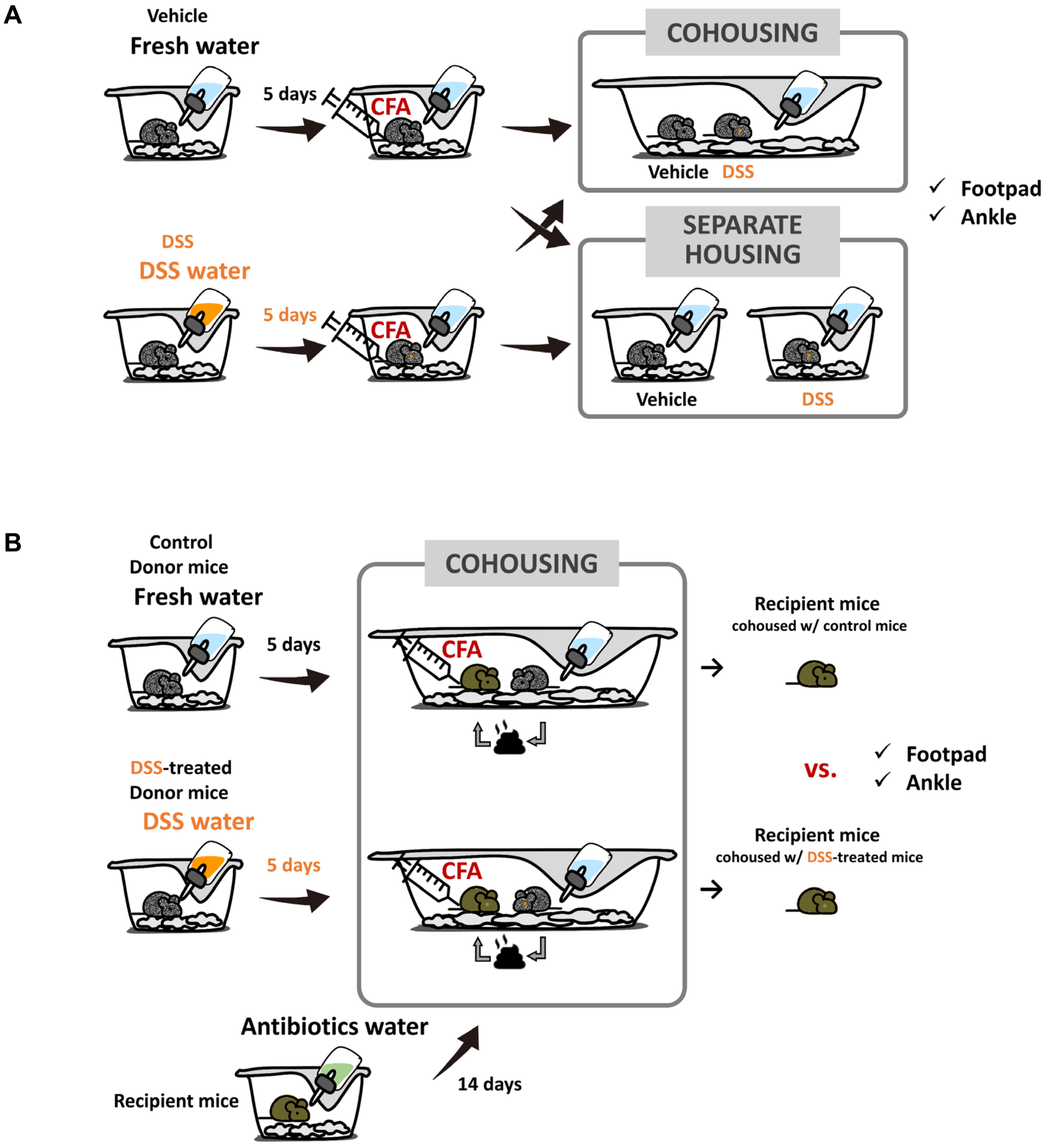

Figure S2 Experimental diagrams about comparing cohousing and separate housing and about transferring gut microbes. (A) Mice were provided with fresh water or DSS water bottles for 5 days. Upon the cessation of the DSS-treatment, CFA and IFA were subcutaneously injected into each footpad of all the mice, and the control and colitis groups were raised in the same cages or separately raised in divided cages. (B) Mice were divided into donor and recipient groups. The recipient group was provided with water bottles containing an antibiotic cocktail for 2 weeks before cohousing. The donor mice were divided into vehicle and DSS groups. The mice in each donor group were cohoused with the recipient mice after 5 days of the DSS treatment. Two days later, CFA and IFA were injected into the footpads of the recipient mice. Footpad and ankle swellings in the CFA-injected limbs were measured using calipers.

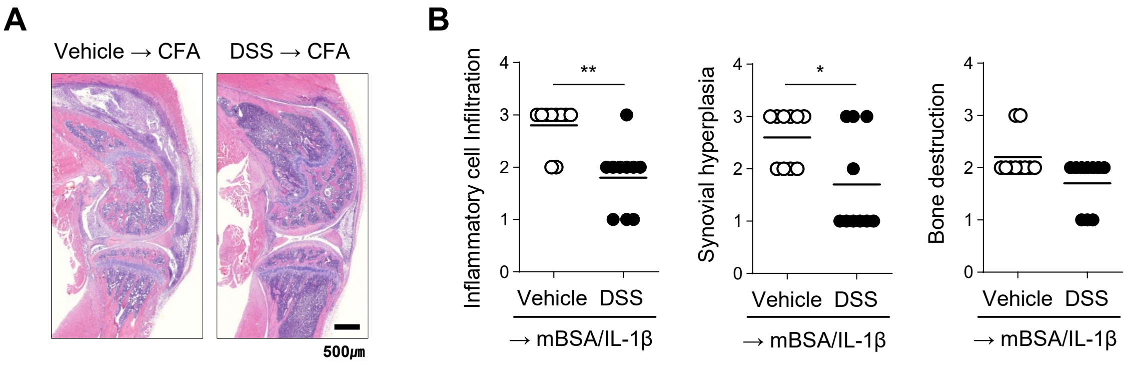

Figure S3 Protective effects of colitis on mBSA/IL-1β-induced arthritis. (A) Shown are examples of H&E-stained knee joint section of the mBSA/IL-1β-challenged limbs (after 7 days of mBSA injection). Scale bar, 500 μm. (B) Severity of synovial hyperplasia, inflammation, and bone destruction scored using a scoring system described in Ref. 4 (Supplemental material). Each dot represents an individual mouse and the means are displayed as a line. *P < 0.05 and ** P < 0.01 by the Mann-Whitney test (B).

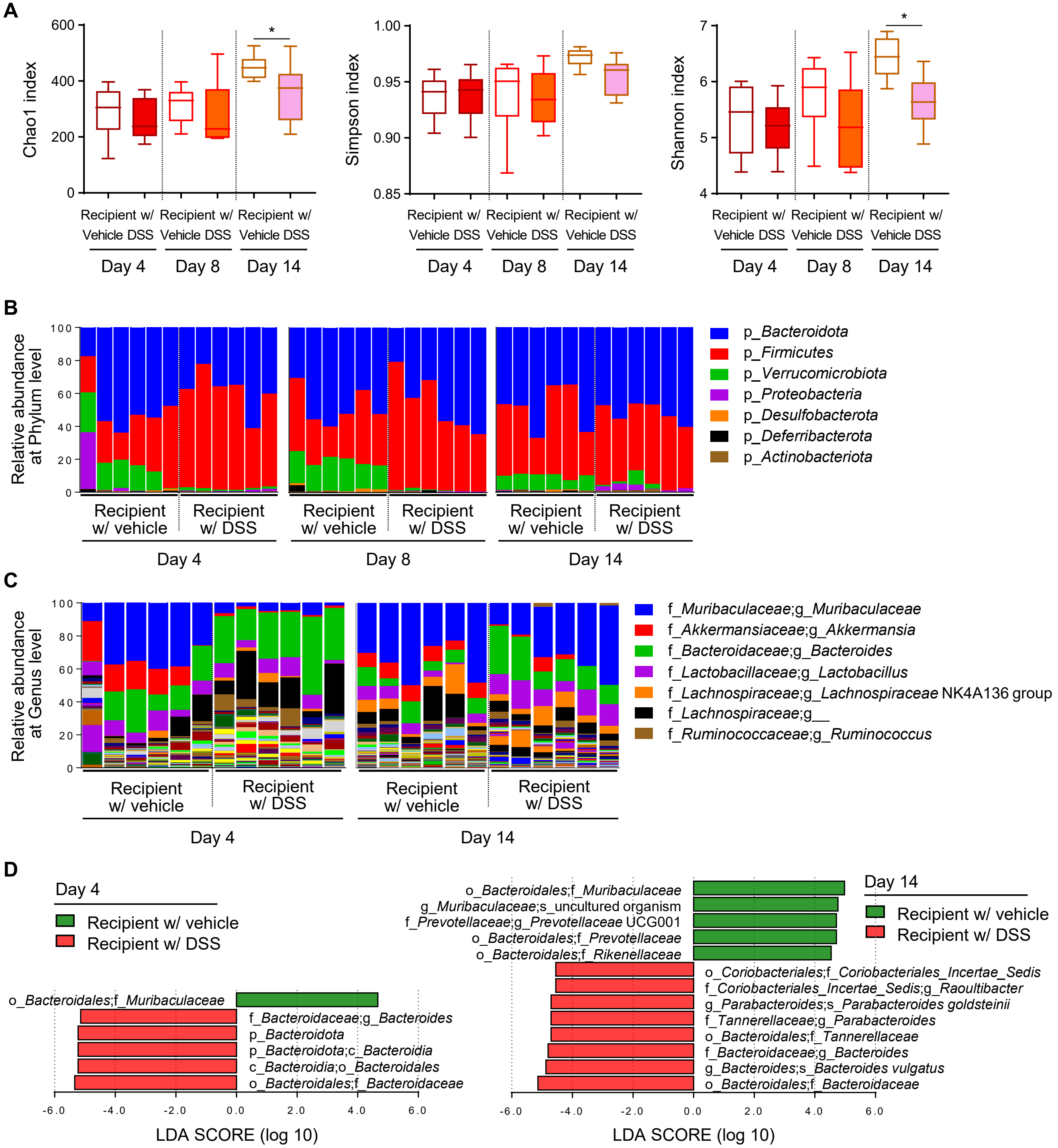

Figure S4 Gut microbial alteration of recipient mice cohoused with DSS-treated mice. (A) Comparison of alpha-diversities in the gut microbial composition of recipient mice (on days 4, 8, and 14). (B, C) Relative abundance of bacterial taxa at the phylum (B) and genus (C) levels. The top taxa are listed in the legend. (D) LEfSe analysis of microbial composition of recipients (on days 4 and 14) cohoused with vehicle and DSS-treated groups (logarithmic LDA score > 4). *P < 0.05 by the Mann-Whitney test (A).

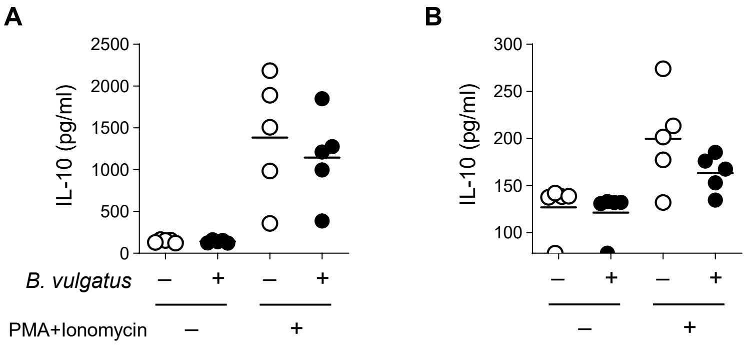

Figure S5 IL-10 expression in the splenocytes and lymphocytes of *B. vulgatus*-administered mice. Spleen and lymph nodes were obtained from mice inoculated with PBS or *B. vulgatus* (on day 0 in the Figure 4A). The splenocytes (A) and lymphocytes (B) were treated with PMA and ionomycin for 24 h. IL-10 was measured in the culture supernatants. Each dot represents an individual mouse and the means are displayed as a line.

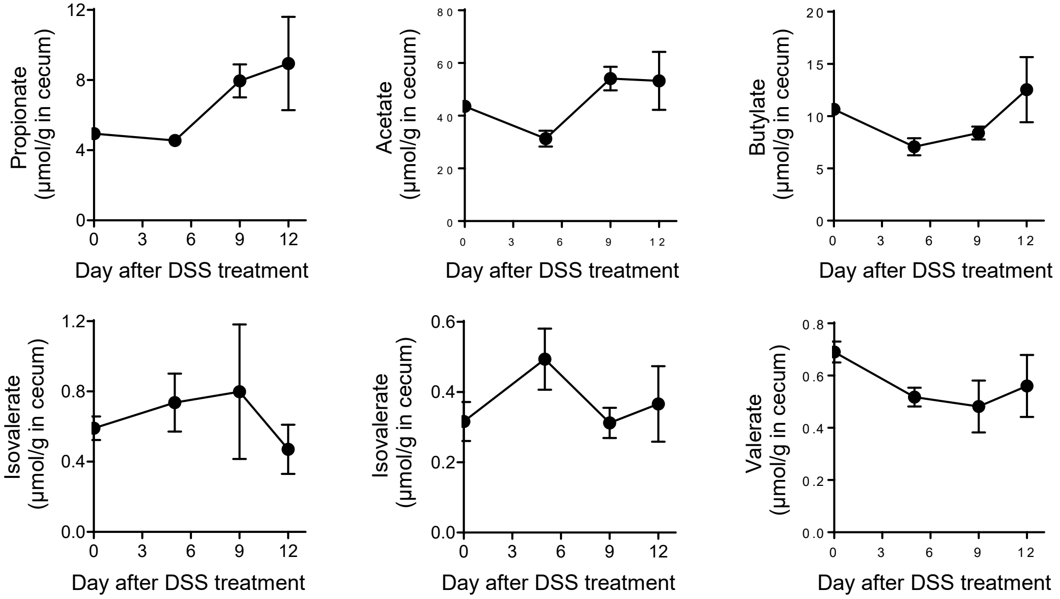

Figure S6 Kinetics of short-chain fatty acids in the cecum contents. The amount of SCFAs in the cecum contents (*n* = 9 per each time point) collected at indicated time points. The data are shown as means ± SEM.

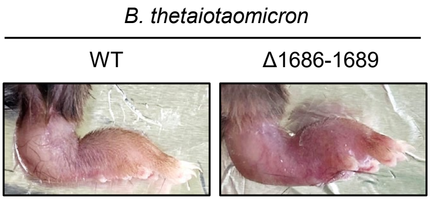

Figure S7 Propionate-producing *B. thetaiotaomicron* ameliorates CFA-induced arthritis. Mice were administered with *B. thetaiotaomicron* (WT or mutant Δ1686-1689). Shown are examples of macroscopic pictures of the CFA-challenged paws (on day 14 in Figure 4).

Table S1 Disease activity index (DAI) score used to evaluate the DSS-induced colitis. The DAI value is calculated as the sum of the scores for body weight loss, blood in stool and stool consistency.

Score	Body weight loss (%)	Visible blood in stool	Stool consistency
**0**	≤ 0	Normal	Normal
**1**	0 – 5	–	–
**2**	5 – 10	Slight bleeding	Loose stool
**3**	10 – 15	–	–
**4**	15 – 20	Gross bleeding	Diarrhea
**5**	> 20	–	–
